# Allometry and integration do not strongly constrain beak shape evolution in large‐billed (*Corvus macrorhynchos*) and carrion crows (*Corvus corone*)

**DOI:** 10.1002/ece3.4440

**Published:** 2018-09-21

**Authors:** Takeshi Yamasaki, Sou Aoki, Masayoshi Tokita

**Affiliations:** ^1^ Division of Natural History Yamashina Institute for Ornithology Abiko Chiba Japan; ^2^ Department of Biology Faculty of Science Toho University Funabashi Chiba Japan

**Keywords:** allometry, avian beak, evolutionary constraints, geometric morphometrics, integration, selection pressures

## Abstract

A recent geometric morphometric study on certain landbird lineages revealed that a major part of the variation in beak shape is accounted for by skull size and cranial shape. The study interpreted this result as evidence for the presence of strong evolutionary constraints that severely prevented beak shape from evolving substantially away from predictions of allometry and morphological integration. However, there is another overlooked but similarly plausible explanation for this result: The reason why beak shape does not depart much from predictions might simply be that selection pressures favoring such changes in shape are themselves rare. Here, to evaluate the intensity of evolutionary constraints on avian beak shape more appropriately, we selected large‐billed (*Corvus macrorhynchos*) and carrion crows (*Corvus corone*) as study objects. These landbird species seem to experience selection pressures favoring a departure from an allometric trajectory. A landmark‐based geometric morphometric approach using three‐dimensional reconstructions of CT scan images revealed that only 45.4% of the total shape variation was explained by allometry and beak–braincase integration. This suggests that when a selection pressure acts in a different direction to allometry and integration, avian beak shape can react to it and evolve flexibly. As traditionally considered, evolutionary constraints on avian beak shape might not be all that strong.

## 1 INTRODUCTION

Avian beak shape, one of the main and long‐standing study objects of evolutionary biology, has generally been regarded as representing evolutionary adaptations to feeding ecology (e.g., Grant & Grant, 1993; Jønsson et al., 2012; Lovette, Bermingham, & Ricklefs, 2002). However, this traditional view was recently challenged by a geometric morphometric study. Bright, Marugán‐Lobón, Cobb, and Rayfield (2016) examined birds‐of‐prey, a basal lineage of the landbird radiation, and revealed that almost 80% of the interspecific variation in skull shape, including the beak, was accounted for by allometry and beak–braincase integration. According to their interpretation, this result suggests that the evolution of beak shape away from predictions of allometry and integration in landbirds, even if it is adaptive, might be almost prohibited by the presence of strong evolutionary constraints.

This hypothesis is appealing, in that it provides an explanation for the fact that many avian lineages do not experience adaptive radiation, even when encountering ecological opportunities. Under this hypothesis, lineages which have undergone adaptive radiation such as Darwin's finches, Hawaiian honeycreepers, and vangas are regarded as groups that have exceptionally broken the genetic lock. However, it should be noted that this hypothesis is based on an unproven assumption. Bright et al. (2016) a priori postulated the presence of selection pressures acting in a different direction from allometry and integration, although they did not have any evidence for these hypothesized selection pressures. If such selection pressures actually existed, the strong association between beak shape and skull size/cranial shape that has been detected would mean the presence of strong evolutionary constraints (i.e., processes preventing or slowing down evolutionary optimization of a trait; Pélabon et al., 2014). However, if selection pressures acting in a different direction to allometry and integration were absent, one would not expect to detect nonstandard beak shapes. In such cases, there would be no necessity for supposing strong evolutionary constraints.

The intensity of evolutionary constraints preventing deviations from allometry and integration trends should be estimated using species that experience selection pressures favoring such deviations. The large‐billed (*Corvus macrorhynchos*) and carrion crows (*Corvus corone*) selected as our study objects are examples of such species.

The large‐billed crow has a broad distribution, breeding from Afghanistan to Southeast Asia and East Asia (Madge, 2017). This distribution overlaps with that of the carrion crow in a relatively small area, which includes northeastern Eurasia, Sakhalin, and the Japanese archipelago (The Ornithological Society of Japan, 2012). Within the sympatric range, the body size of the large‐billed crow is always larger than that of the carrion crow (Tamada, 2004). In corvids, larger skulls are known to be related to a relatively longer bill and more lateral‐facing orbits (Kulemeyer, Asbahr, Gunz, Frahnert, & Bairlein, 2009). Thus, from the viewpoint of allometric constraints, when the large‐billed crow is in sympatry with the carrion crow, it is expected that the aforementioned characteristics will be more pronounced than in the carrion crow.

In contrast, differences in feeding ecology between these sympatric species lead to the opposite expectation. Within the sympatric range, the large‐billed crow commonly drops to take food spotted from a vantage point (Matsubara, 2003). This visually guided foraging leads almost exclusively to pecking, a method characterized by picking at a food item at the surface (Kulemeyer et al., 2009). In contrast, the carrion crow commonly forages for food items while walking on the ground (Matsubara, 2003). In this foraging behavior, probing, which is defined as feeding on a food item below the surface (Kulemeyer et al., 2009), becomes an effective feeding technique. Morphologically, a relatively short bill and more forward‐facing eyes that facilitate binocular vision over the bill‐tip and help to keep food objects in sight are better suited to pecking (Kulemeyer et al., 2009), while probing is thought to require a smaller field of binocular vision. Thus, from the viewpoint of feeding ecology, the large‐billed crow in sympatry is expected to undergo a selective pressure favoring a relatively shorter bill and more forward‐facing orbits than the carrion crow.

In this study, we also examined the large‐billed crow living in allopatry. In comparison with the sympatric population of the same species, the allopatric population shows a smaller body size, more like that of the carrion crow (Meinertzhagen, 1926). In addition, the allopatric population is also known to show ecological characteristics rather similar to those of the carrion crow (Higuchi, 1979). Questions can therefore be asked as to whether the allometric constraints of its large size have prevented the large‐billed crow in sympatry from evolving a beak and cranial shape suited to pecking, or whether it has been released from such constraints and undergone adaptations in skull shape. To answer these questions, we conducted three‐dimensional (3D) geometric morphometric analysis of computed tomography (CT) images of large‐billed and carrion crows from sympatric populations and large‐billed crows from allopatric populations.

## 2 MATERIALS AND METHODS

### 2.1 Samples

A total of 39 adult male skulls collected from East Asian islands were examined: 12 of the large‐billed crow living in sympatry, 7 of the carrion crow living in sympatry, and 20 of the large‐billed crow living in allopatry.

**Figure 1:**
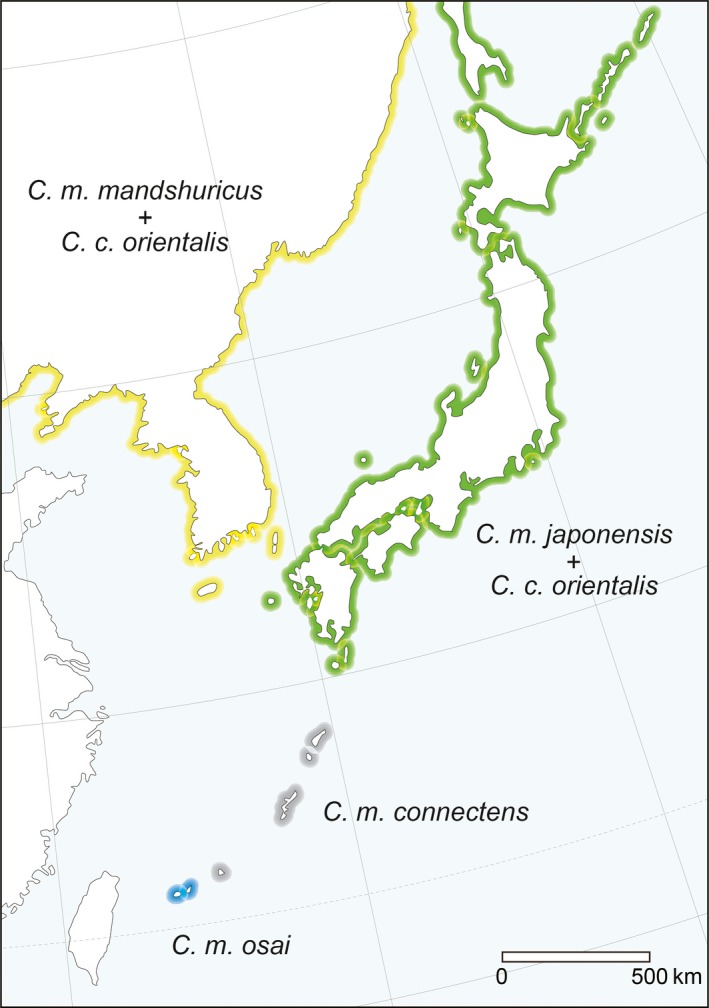
A distribution map of the examined subspecies of the large‐billed (*Corvus macrorhynchos*) and carrion crows (*Corvus corone*) in East Asia

The sample of the sympatric population of the large‐billed crow consisted of the two subspecies *C. m. japonensis* (10 specimens) and *C. m. mandshuricus* (2 specimens), while the allopatric sample consisted of the two subspecies *C. m. connectens* (10 specimens) and *C. m. osai* (10 specimens). The carrion crow sample included only a single subspecies, *C. c. orientalis*. All specimens were deposited at the Yamashina Institute for Ornithology (see Figure 1 and Appendix for details).

### 2.2 CT scanning and 3D reconstruction of skulls

Computed tomography scanning was performed using a LaTheta LCT100™ micro‐CT scanner (Hitachi Ltd., Tokyo, Japan) with the parameters set to 50 kV, 1 mA, and a slice thickness of 200 μm, thus allowing high‐resolution 3D images of the skull samples to be obtained. Amira 5.2.2 or 5.4.5 software (FEI Visualization Sciences Group, Burlington, MA, USA) was used to reconstruct 3D images from the scanned cross sections.

### 2.3 Landmarks and semilandmarks

**Figure 2:**
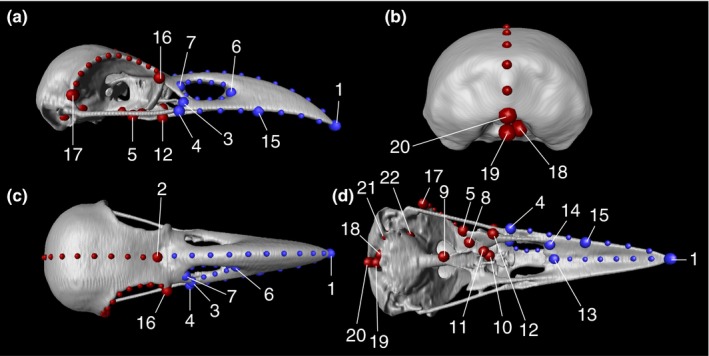
The landmarks and semilandmarks used illustrated on a skull. (a) Lateral view. (b) Caudal view. (c) Dorsal view. (d) Ventral view. Large dots are landmarks and small dots are semilandmarks. They are partitioned into two blocks: beak (blue) and braincase (red). The numbers indicate each landmark employed in the analyses. Definitions of the landmarks are given in Table 1

We placed a total of 22 landmarks (LMs) onto the right side of each 3D skull image using Amira (Table 1 and Figure 2). The skulls used were largely symmetrical, with the evolution of cranial left–right asymmetry not being addressed in this study. This procedure allowed us to increase the number of specimens examined, because it enabled the inclusion of specimens whose left side was damaged.

**Table 1:** Definitions of the landmarks and their allocation to beak and braincase blocks

Number	Definition	Block
1	Beak tip	Beak
2	Midpoint of craniofacial hinge	Braincase
3	Maximum of curvature at the rostral end of the Fossa et Fenestra antorbitalis	Beak
4	Maximum of curvature at the lateral intersection of the Processus maxillaries and the jugal	Beak
5	Most posterior point of the Os palatinum	Braincase
6	Maximum of curvature at the rostral end of the external nares	Beak
7	Maximum of curvature at the caudal end of the external nares	Beak
8	Maximum of curvature at the posterior site of the Os palatinum	Braincase
9	Joint point between palatine and pterygoid	Braincase
10	Tip of the process of interior Os palatin	Braincase
11	Maximum of curvature at the anterior site of the Os palatinum	Braincase
12	Maximum of curvature at the outer Os palatinum	Braincase
13	Most caudal point of ventral central line on beak	Beak
14	Inner junction of palatine and premaxilla	Beak
15	Outer junction of palatine and premaxilla	Beak
16	Most dorsolateral point of the Os lacrimale	Braincase
17	Most distal point of the process of postorbital bone	Braincase
18	Maximum of curvature at the edge of Foramen magnum	Braincase
19	Tip of the process at anterior edge of Foramen magnum	Braincase
20	Most posterior point of the foramen magnum	Braincase
21	Ostium canalis ophthalmici externi	Braincase
22	Foramen n. maxillomandibularis	Braincase

As the dermal (intramembranous) bones that make up the skull (e.g., frontal, parietal, maxilla, and premaxilla bones) are tightly fused to each other in birds, it can be difficult to select a large number of landmarks along sutures (Bright et al., 2016; Kulemeyer et al., 2009; Tokita, Yano, James, & Abzhanov, 2017). To overcome this problem, we also used 54 semilandmarks. These semilandmarks were initially set at equal spacings along each of the following seven anatomical curves: (1) LM1–LM2, (2) LM2–LM20, (3) LM16–LM17, (4) LM6–LM7 (the upper curve), (5) LM6–LM7 (the lower curve), (6) LM1–LM13, and (7) LM1–LM4 (Figure 2). The *xyz* coordinate values for these equidistant semilandmarks were obtained according to the method of Morita, Ogihara, Kanai, and Suzuki (2013). Using Amira, we first plotted 4–11 points (guide points) at roughly equal spacings on each of the anatomical curves (10 points for curves 1 and 3, 11 points for curve 2, 4 points for curves 4 and 5, 6 points for curve 6, and 7 points for curve 7). We then fitted a seventh (for anatomical curves 4 and 5) or ninth‐order (for the other curves) Bézier curve to these guide points. To determine the parameter values for these Bézier curves and to select points on them minimizing the sum of the squared distances between the said points and corresponding guide points, we performed a numerical optimization using the NLPQN subroutine of the SAS IML procedure (SAS 9.3 and 9.4; SAS, Cary, NC, USA). To shorten the calculation time, we adopted an additional nonlinear constraint: Vectors from the guide points to the corresponding points had to be perpendicular to the tangent vectors of the Bézier curves. Lastly, to obtain exactly equally spaced semilandmarks, we calculated lengths along the optimized Bézier curves using a numerical integration procedure, the QUAD subroutine of the SAS IML procedure. For each of the anatomical curves, we selected as many semilandmarks as there were guide points.

The equidistant semilandmarks were then allowed to slide along the curves, as the equidistance could result in possible artifacts in the representation of morphological variability (Gunz & Mitteroecker, 2013). Using the SAS IML procedure and its NLPQN subroutine, we calculated the positions of the sliding semilandmarks that minimized the sum of the squared Procrustes distances obtained in a full ordinary Procrustes analysis (OPA; Dryden & Mardia, 2016) of the landmarks and semilandmarks.

### 2.4 Geometric morphometrics

The full OPA yielded a matrix of shape coordinates from which all information related to position, scale, and orientation had been removed. The analysis also provided a centroid size for each specimen, which we used as an indicator of the overall size of the skull.

To describe the size‐related shape variation (i.e., allometry), we conducted a multivariate regression of the *xyz* components of the shape coordinates against the centroid sizes, using the method of Khattree and Naik (1999). To confirm statistical significance, we performed a permutation test (10,000 iterations) using the SAS IML procedure. We used the sum of squares predicted by the regression as the test statistic. We also used the same SAS procedure to calculate regression scores (Drake & Klingenberg, 2008) to evaluate the strength of the association between size and shape. The detected pattern of allometric transformation was then visualized using the prediction from the regression and the LandmarkSurfaceWarp module of Amira.

Next, we conducted shape comparisons after removing the allometric effects of size. We conducted a principal component analysis (PCA) on the regression residuals using the SAS PRINCOMP procedure. We also tested the multivariate mean differences in the residuals using nonparametric multivariate analysis of variance based on Euclidean distances (NPMANOVA; Anderson, 2001), with the calculations being performed using the SAS IML procedure. NPMANOVA was conducted for (a) a comparison between the two sympatric species and (b) a comparison between the two populations of the large‐billed crow, with the significance level being adjusted to 0.025 (Bonferroni correction for multiple comparisons). We further performed a series of two sample univariate Satterthwaite *t* tests of the residuals to compare (c) the two species in sympatry and (d) the sympatric and allopatric populations of the large‐billed crow. For these calculations, we used the SAS TTEST procedure, with the significance level again being adjusted to 0.025.

We visualized the results of the *t* tests using the following four‐step method. (a) We first calculated the shape coordinates of a hypothetical mean skull that was allometrically predicted from the average centroid size of all specimens. (b) We then calculated sample‐means for each of the residuals showing significant differences in the *t* tests (*p* < 0.025). (c) We added the sample‐means for each sample separately to the corresponding xyz components of the shape coordinates of the hypothetical mean skull. For emphasis, each of the sample‐means was multiplied by three before the addition. (d) Using the resulting coordinates and the Amira LandmarkSurfaceWarp module, we graphically expressed the skull shape differences detected by the *t* tests. The graphics depicted using this procedure show how and where the skull shapes differ between the two groups. They are essentially equivalent to the tables frequently used to summarize the results of univariate analyses in linear morphometric studies.

To analyze the effect of morphological integration in the absence of the allometric effect, we partitioned the landmarks and semilandmarks into two blocks representing the beak and the braincase (Table 1 and Figure 2). We calculated the RV index of integration (Klingenberg, 2009) from the residuals of the allometric regression using the SAS COLL and IML procedures. The statistical significance of integration was then assessed using a permutation test (250 iterations), in which we randomly shuffled combinations of beak and braincase blocks, using the RV index as the test statistic. Then, to condense the covariation information into a few variables, we conducted a two‐block partial least square (PLS) analysis (Rohlf & Corti, 2000) using the SAS COLL, STANDARD, and IML procedures. We assessed the significance of each of the PLS axes using a permutation test (10,000 iterations), as proposed by Rohlf et al. (2000).

To determine the extent to which the overall shape variation was explained by allometry, we calculated the explained sum of squares divided by the total sum of squares (i.e., the coefficient of determination, *R* ^2^). We then evaluated the extent of the overall shape variation explained by the nonallometric covariation between the beak and the braincase according to the method described by Bright et al. (2016). We first performed a regression of the nonallometric PLS1 scores for the braincase block against those for the beak block. The prediction from this regression was then used as an independent variable in an additional regression analysis of the nonallometric shape data. We calculated the ratio of the explained sum of squares of this regression divided by the total sum of squares of the *original* Procrustes coordinates (not the nonallometric shape data). We also calculated these two ratios for (a) data with *C. corone* removed and (b) the average Procrustes coordinates and centroid size for the island populations of the two species.

## 3 RESULTS

**Figure 3:**
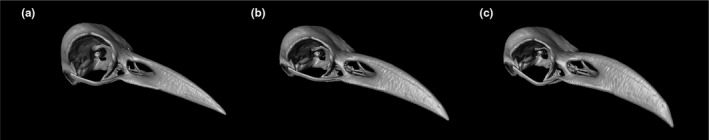
Allometric changes in skull shape in *Corvus* species. Skull shapes predicted for −3 (a), 0 (b), and +3 (c) standard deviations from the all‐specimen mean centroid size

**Figure 4:**
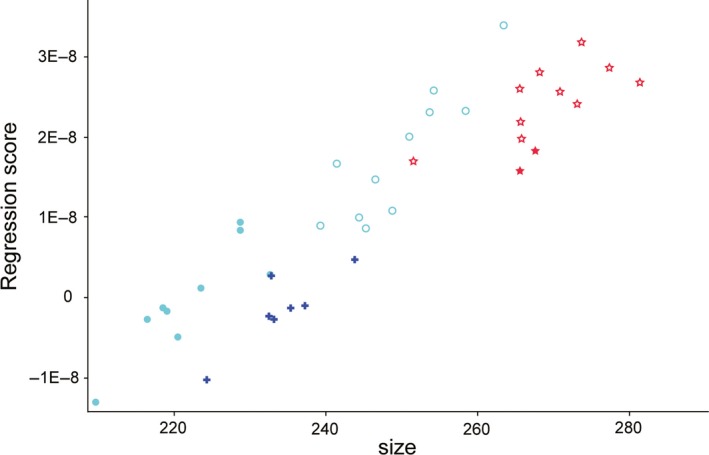
Regression scores plotted against skull centroid size. Stars represent the large‐billed crow in sympatry (open stars for *Corvus macrorhynchos japonensis*; filled stars for *C. m. mandshuricus*), crosses the carrion crow in sympatry, and circles the large‐billed crow in allopatry (open circles for *C. m. connectens*; filled circles for *C. m. osai*)

In the combined data including all specimens, we detected a tendency for larger skulls to have relatively long and curved bills and more lateral‐facing orbits (Figure 3). The permutation test showed that this allometry was statistically significant (*p* < 0.0001). The scatterplot of the regression scores on the centroid size also showed a somewhat linear pattern (Figure 4). However, the association between size and shape was not very strong: Only 23.8% of the shape variation could be predicted by size.

**Figure 5:**
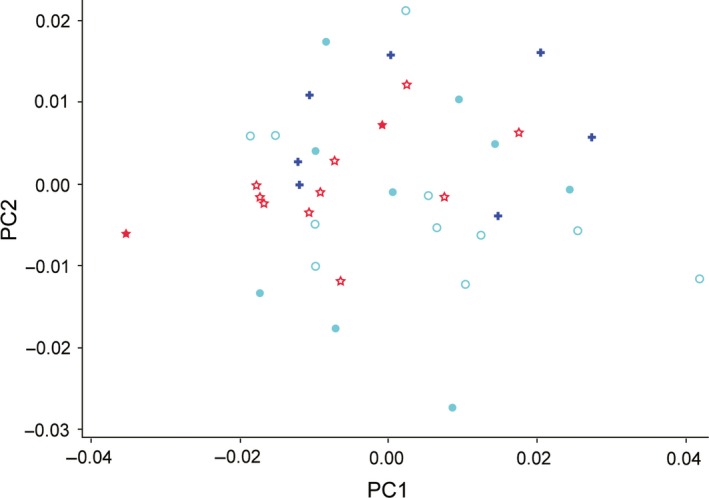
Scatterplot of the first two factors of the principal component analysis on the nonallometric component of skull shape variation. For symbols, see the legend of Figure 4

In the PCA of the regression residuals, the first six components accounted for 73.6% of the total variation. However, the scatterplots of these components (Figure 5 and Figure S1) revealed that the ranges of the three samples overlapped considerably. Therefore, this analysis could not confirm a tendency for the large‐billed crow to have a skull shape suited to pecking when in sympatry.

**Figure 6:**
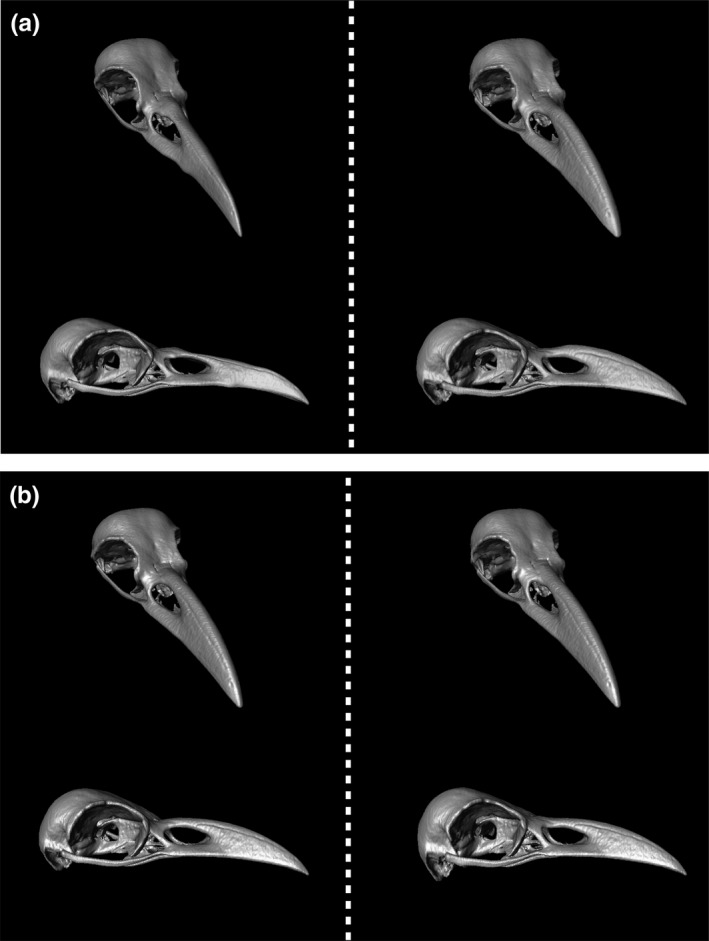
Graphical summary of two sample *t* tests for the skull shape comparisons taking allometric effects into account. Each surface representation is based on the following formula: (the allometric expectation predicted from the all‐specimen mean of centroid size) + 3 × [residual means of the focal population with significant differences from the counterpart (*p* < 0.025)]. (a) Comparisons of the large‐billed crow (right) and carrion crow (left) in sympatry. Upper and lower drawings represent the skull in oblique front and right lateral views, respectively. (b) Comparisons of the sympatric (right) and allopatric (left) populations of the large‐billed crow. Upper and lower drawings represent the skull in oblique front and right lateral views, respectively

In the comparison between the two sympatric species, the NPMANOVA detected a significant multivariate mean difference in the regression residuals (*p* = 0.0237). The two sample *t* tests of the residuals detected significant differences in 43 coordinate components (out of a total of 222; *p* < 0.025). According to the graphical representation of the *t* test results (Figure 6 and Animation S1), there was no difference in bill length between the large‐billed crow and carrion crow in sympatry. However, the large‐billed crow had a more enlarged braincase and thus a proportionally shorter bill than the carrion crow. Moreover, the braincase of the large‐billed crow was almost as wide as that of the carrion crow at the rostral end of the orbits, but wider at the caudal end. Consequently, the orbits of the large‐billed crow in sympatry were more forward‐facing than those of the Carrion Crow.

In the comparison between the two populations of the large‐billed crow, the NPMANOVA did not detect any significant multivariate mean difference in the regression residuals (*p* > 0.025). However, this result might not be conclusive, owing to the small sample size of our data and the relatively low *p*‐value (*p* = 0.0632). The two sample *t* tests of the residuals showed significant differences (*p* < 0.025) in 12 coordinate components (out of a total of 222). The graphical representation of the results of the *t* tests (Figure 6 and Animation S2) revealed that the two populations did not differ from each other in bill length, although the sympatric population had orbits with more concave margins toward the midline, and dorsoventrally, more expanded caudal bony walls. As a result, the orbits of the sympatric populations were more forward‐facing than those of the allopatric population.

**Figure 7:**
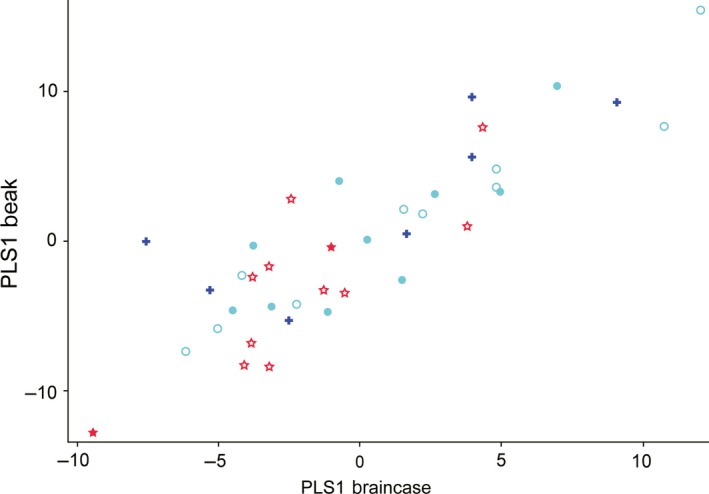
Scatterplot of the first pair of the partial least square analysis based on the beak and braincase landmark blocks. For symbols, see the legend of Figure 4

The beak–braincase covariance was statistically significant (RV = 0.59; permutation test, *p* < 0.004). In the PLS analysis, the first five axes represented 67.7%, 15.4%, 4.0%, 3.8%, and 2.7% of the covariation, respectively. The permutation tests showed that only the first axis was significant (*p* = 0.0013 for PLS1; *p* > 0.05 for the other four). The scatterplot of PLS1 (Figure 7) also showed a high degree of correlation between beak and braincase blocks (correlation = 0.87).

We estimated that 23.8% of the total variation in skull shape was explained by allometry, with the amount of integrated variation that was independent of allometry being calculated as 21.6%. Therefore, the evolutionary constraints of allometry and integration predicted 45.4% of the total shape variation. The total effect of allometry (24.6%) and integration (24.7%) constraints accounted for 49.4% of the total shape variation in *C. macrorhynchos* samples alone. As for the island populations of the two species, the allometry and integration constraints explained 29.2% and 17.0% of the total variation, respectively (46.2% in total).

## 4 DISCUSSION

### 4.1 Evolutionary constraints on beak and cranial shape

Our results confirmed a tendency for larger skulls to possess a relatively longer bill and more lateral‐facing orbits (Figure 3; permutation test, *p* < 0.0001), as reported by Kulemeyer et al. (2009) for European corvids. Judging from this allometry, the sympatric population of the large‐billed crow, which had the largest body size in our samples (Madge, 2017; Meinertzhagen, 1926; Tamada, 2004), would be expected to show such morphological features most clearly. However, as mentioned in the introduction, this sympatric population frequently uses pecking as a foraging technique, and skulls with a relatively short bill and more forward‐facing orbits are considered better suited to pecking (Kulemeyer et al., 2009). The following question was therefore posed “has the large‐billed crow in sympatry with the carrion crow evolved a skull for pecking, despite allometric constraints”?

To answer this question requires an examination of shape variation from which the allometric effects of size have been removed. A straightforward and often used method is a PCA of the residuals of a multivariate regression of shape coordinates on centroid size (e.g., Bright et al., 2016; Klingenberg & Marugán‐Lobón, 2013; Tokita et al., 2017). However, this method seems inappropriate for the present study; in this study, we are interested in relative bill length and orbit orientation, both of which were revealed to be highly dependent on size (Figure 3). Such components tend to give small residuals, and thus, we must examine whether there are subtle but substantial among‐sample differences in these small residuals. However, such residuals are expected to have little impact on the results of a PCA, because they would show only small variances. In fact, a PCA of our data did not indicate any substantial shape differences among the samples (Figure 5 and Figure S1). Canonical variate analysis (CVA) seems a promising alternative, but our data are unbalanced in that the number of predictor variables (222 residuals) considerably exceeds the sample size of the smallest group (7 specimens in *C. corone*). Such imbalance generally gives inappropriate results in CVA (“overfitting”; Tabachnick & Fidell, 2014), and therefore, we had to search for another more appropriate approach. In this study, we conducted Bonferroni corrected NPMANOVA (Anderson, 2001) to test for multivariate mean differences (ordinary MANOVA cannot be performed due to insufficient error degrees of freedom) and performed direct comparisons of the residuals using Bonferroni corrected univariate *t* tests.

The NPMANOVA detected a significant multivariate mean difference in the residuals between the two sympatric species (*p* = 0.0237). The results of the comparison with the *t* tests are graphically summarized in Figure 6 and Animation S1, and they clearly illustrate that the large‐billed crow in sympatry has evolved a skull shape better suited to pecking than that of the sympatric carrion crow. In addition, although we have to keep in mind that no significant difference was found in the multivariate residuals (NPMANOVA, *p* = 0.0632), the *t* tests comparing the two populations of the large‐billed crow (Figure 6 and Animation S2) seem to suggest a tendency for the sympatric population to have a skull shape better suited to pecking than that of the allopatric population.

The association between skull size and shape in our data was weak, despite being significant (permutation test, *p* < 0.0001), with the allometry explaining only 23.8% of the total variation. This is in contrast to the result of a recent study on birds‐of‐prey (Bright et al., 2016), in which 47.5% of the total variation in skull shape was explained by allometry.

Our data also revealed that the integration between the beak and braincase was significant (permutation test, *p* < 0.004) and rather strong (Figure 7; correlation = 0.87, RV = 0.59). However, the contribution of this integration to the total shape variation was not very large, with the nonallometric integration accounting for only 21.6% of the total variation, less than the 32.4% estimated for birds‐of‐prey (Bright et al., 2016).

In summary, the evolutionary constraints of allometry and integration were estimated to have a considerably weaker effect on skull shape in our crow dataset than they did in birds‐of‐prey: These constraints accounted for only 45.4% of the total shape variation in our crow dataset, while they accounted for 79.9% of the total shape variation in the birds‐of‐prey (Bright et al., 2016). This conclusion remains essentially unchanged, even when only specimens of the large‐billed crow are considered: Only 49.4% of the total shape variation was explained by the constraints (24.6% by allometry and 24.7% by integration), much less than in the birds‐of‐prey.

We need to keep in mind that our calculation of the ratios was based on only the two crow species. In contrast, the ratios for the birds‐of‐prey were calculated using data from 147 species (Bright et al., 2016). Compared with the raptor dataset, the range of skull sizes covered in our two‐species crow dataset was narrower. Potentially, such paucity of size variation might lead to a lower estimate of the association between shape and size. However, this does not seem a sufficient explanation for the weak association detected in our data, because the large‐billed crow is especially geographically diverse in body size, and our sample included both the largest (*C. m. japonensis*) and the smallest (*C. m. osai*) subspecies. The former's body size overlaps with that of the common raven *C. corax*, while the latter is an island‐dwelling dwarf form with a body size only a little bigger than the western jackdaw *C. monedula* (Meinertzhagen, 1926).

It should also be noted that the present study examined a mixture of infrapopulation, interpopulation, and interspecific variation. Consequently, the allometric constraint we found is a composition of static (infrapopulation) and evolutionary (interpopulation and interspecific) allometry, which also applies to our results for the effect of integration. In contrast, Bright et al. (2016) primarily analyzed the effects of evolutionary allometry and integration (although their dataset also contained some sex‐related variation, unlike ours). To make the comparison between the two studies more appropriate, we re‐evaluated the contributions of allometry and integration constraints to skull shape using averages for the island populations of the two crow species. Again, the result remained similar: 46.2% of the variation in skull shape was explained by the combined effects of allometry (29.2%) and integration (17.0%), which was much smaller than that for the birds‐of‐prey (79.9%; Bright et al., 2016).

In conclusion, our results seem to suggest that when selection pressures act in directions different from the tendencies of allometry and integration, the avian beak can be easily released from these constraints and evolve a shape favored by selection. This supports the traditional view that evolutionary constraints on the avian beak are weak and that the avian beak represents evolutionary adaptations to feeding ecology (e.g., Grant & Grant, 1993; Jønsson et al., 2012; Lovette et al., 2002). The strong association between beak shape and skull size/cranial shape detected in birds‐of‐prey (Bright et al., 2016) might be attributable to the scarcity of selection pressures acting in different directions to the tendencies of allometry and integration.

### 4.2 What prevents dwarfism of the large‐billed crow in sympatry?

Here, we discuss why natural selection favoring a skull shape suited for pecking has not lead to the large‐billed Crow developing a smaller body size in sympatry (Figure 3). Such evolutionary changes in body size are assumed to often occur in birds‐of‐prey in response to changing environmental conditions (Bright et al., 2016). One plausible explanation is that there was another selection force favoring a large body size at work. The large‐billed crow in sympatry commonly drops to take food spotted from a vantage point (Matsubara, 2003). Given that young individuals of the species are known to flock together (Kuroda, 1990), this foraging behavior often leads to situations where many individuals scramble for a food item (see also Soma & Hasegawa, 2003). In such intraspecific competition, larger individuals might have an advantage over smaller ones. In addition, the observation that the large‐billed crow in sympatry often utilizes garbage dumps (Higuchi, 1979) seems to support the idea that this corvid species has a high tendency to scavenge. The stomach contents of the large‐billed crow in sympatry, which include a large amount of meat (Ikeda, 1957; Inukai & Haga, 1953; Nazarov, Trukhin, & Kazikhanova, 1990), also support this idea, although it may also indicate its predatory ability (e.g., Hatakeyama, 2014; Nazarov et al., 1990). In Europe, corvid scavengers are known to exhibit an interspecific dominance hierarchy (Halley, 2001), with dominant species excluding subordinate species from access to carcasses. This hierarchy seems to be determined mainly by body size. Thus, the large‐billed Crow in sympatry might have circumvented dwarfism to retain access to food sources.

In summary, the skull of the large‐billed crow in sympatry seems to have evolved under two forces of natural selection: one that favored a skull shape suited for pecking, and another that favored a large body and thus a large skull. Under the presence of its congeneric competitor, the large‐billed crow might have decreased its dependence on probing, a technique frequently used by the rival Carrion Crow, and instead increased its use of pecking and scavenging (and also possibly predation; see Nazarov et al., 1990 and Hatakeyama, 2014). It is worth noting that large parts of the area where the two species coexist lack breeding populations of the largest corvid scavenger, the common raven. This is quite exceptional in the mid‐ and high latitudes of the Northern Hemisphere (Marzluff, 2017). The absence of this formidable rival might have facilitated the large‐billed crow in becoming a large‐sized corvid scavenger in such areas.

## CONFLICT OF INTEREST

None declared.

## AUTHOR CONTRIBUTIONS

TY and MT contributed to the experimental design, TY collected specimens and acquired CT images, SA established the coordinate data with the assistance of TY and MT, TY performed data analysis and wrote the paper with the assistance of MT. All authors contributed to editing a late version of the paper.

## DATA ACCESSIBILITY

Data available from the Dryad Digital Repository: https://doi.org/10.5061/dryad.tf083ng.

## APPENDIX 1

### 1.1 SPECIMENS EXAMINED

*Corvus macrorhynchos japonensis*: KA004, KA011, KA013, KA022, KA023, KA024, KA031, KA038, KA041, and KA042 from Kagoshima, Kyushu island. *C. m. mandshuricus*: TU004 and TU005 from Tsushima Island. *C. m. connectens*: A003, A013, A016, A017, and A033 from Amamioshima Island; O002, O003, O005, O013, and O014 from Okinawajima Island. *C. m. osai*: IS003, IS012, IS015, IS017, IS022, and IS028 from Ishigakijima Island; KR015 from Kuroshima Island; IRI019, IRI037, and IRI040 from Iriomotejima Island. *C. corone*: YIO71017 from Hokkaido island; YIO71018 from Gifu, Honshu island; B016 from Nara, Honshu island; B022 from Tsushima island; B031 from Miyazaki, Kyushu island; B001 and B005 from Kagoshima, Kyushu island.
