# Supplementary figures and images for: Allometry and integration do not strongly constrain beak shape evolution in large‐billed (Corvus macrorhynchos) and carrion crows (Corvus corone)
Source: Ecol Evol. 2018 Sep 21;8(20):10057–66. doi: 10.1002/ece3.4440 (PMC6206190; doi:10.1002/ece3.4440)

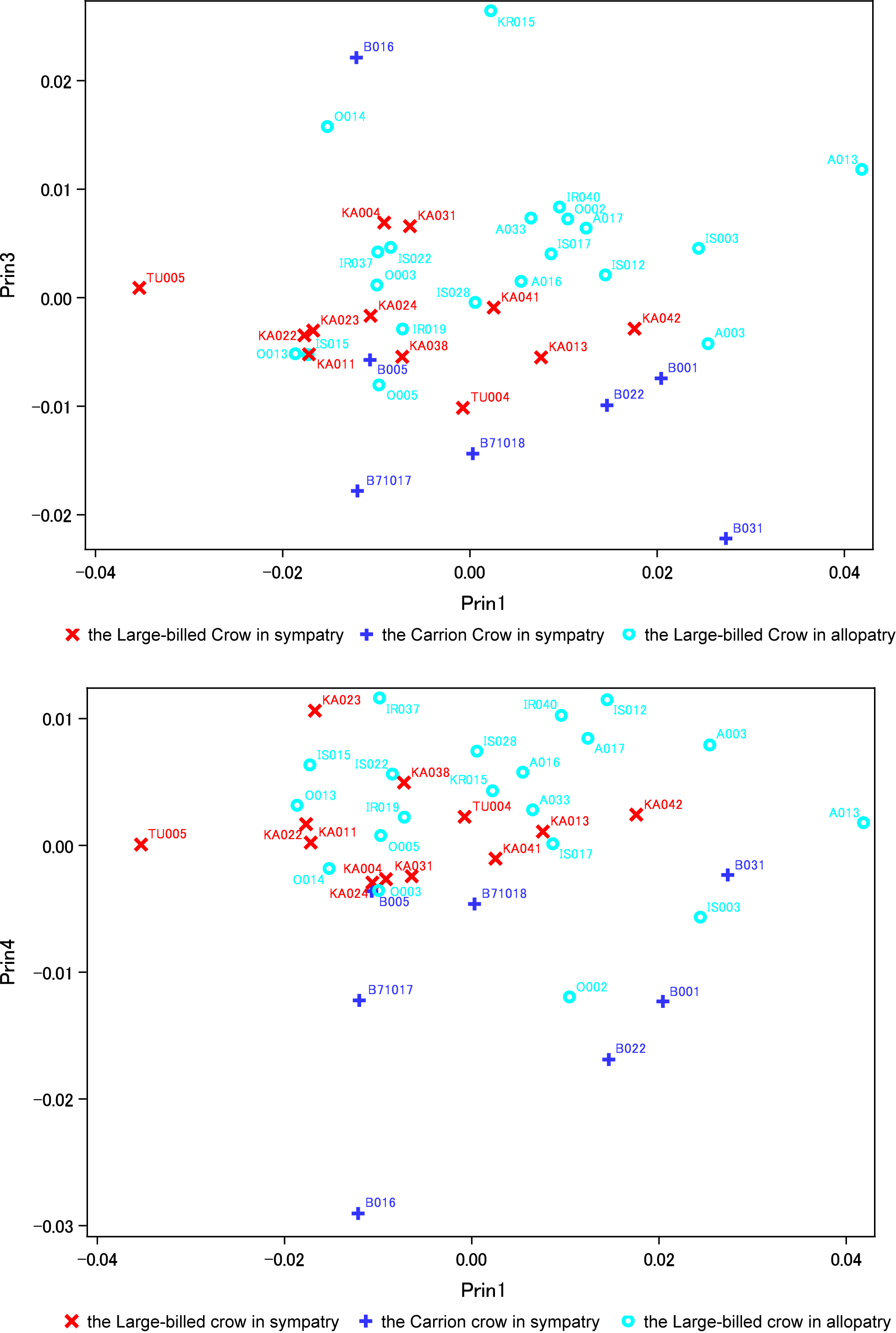

Supplement: Supplementary file 1 [file ECE3-8-10057-s001.jpg]

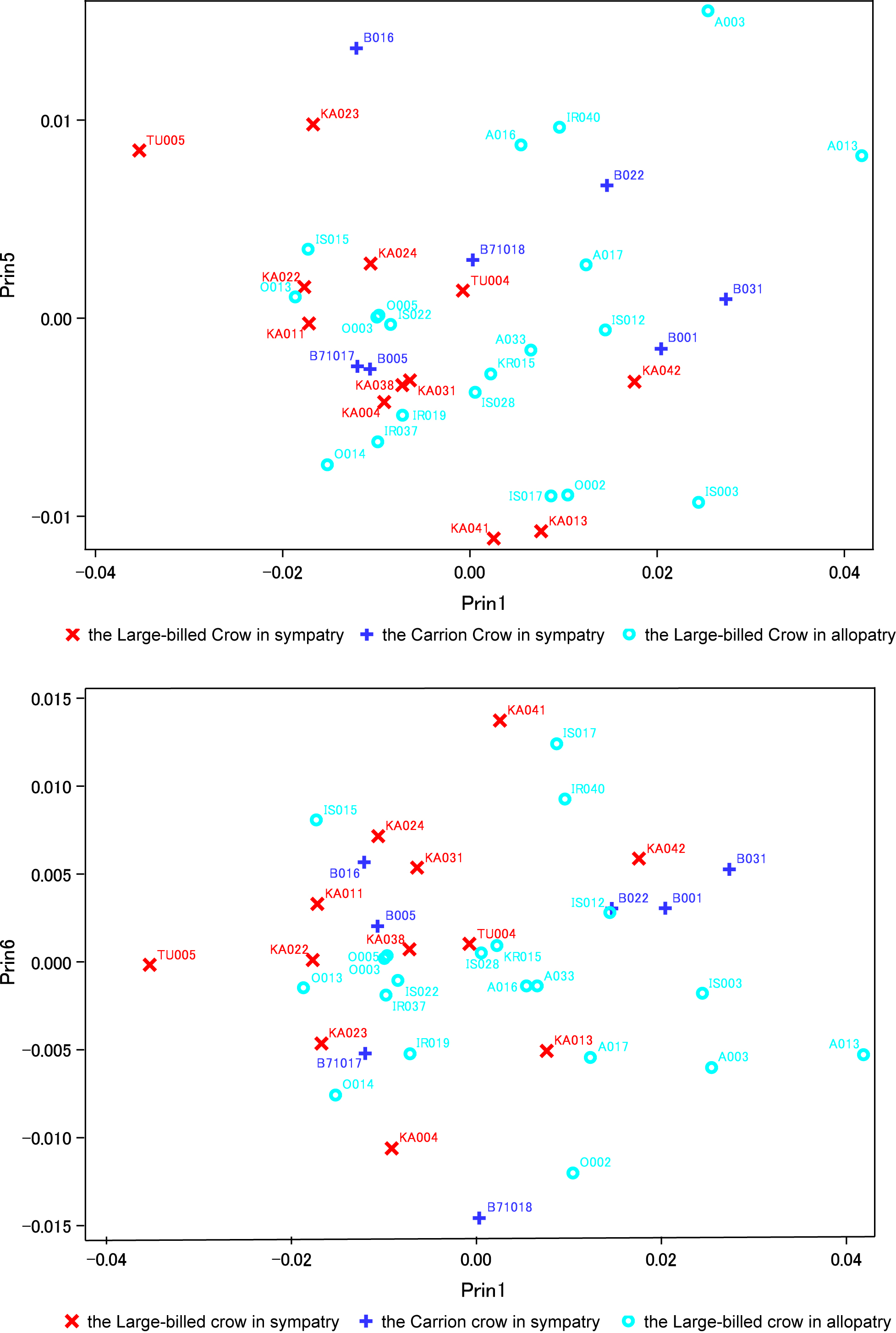

Supplement: Supplementary file 2 [file ECE3-8-10057-s002.jpg]

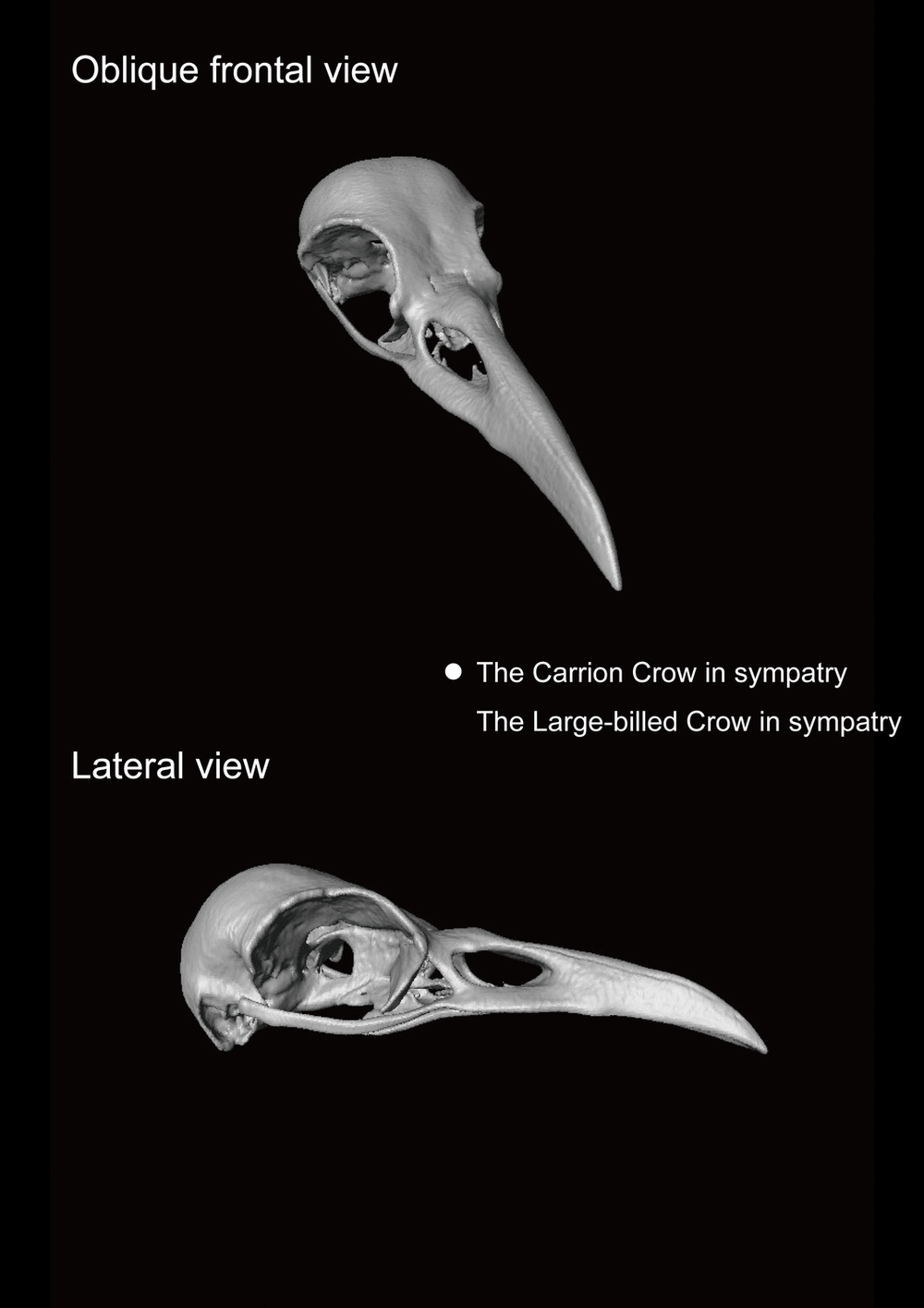

Supplement: Supplementary file 3 [file ECE3-8-10057-s003.gif]

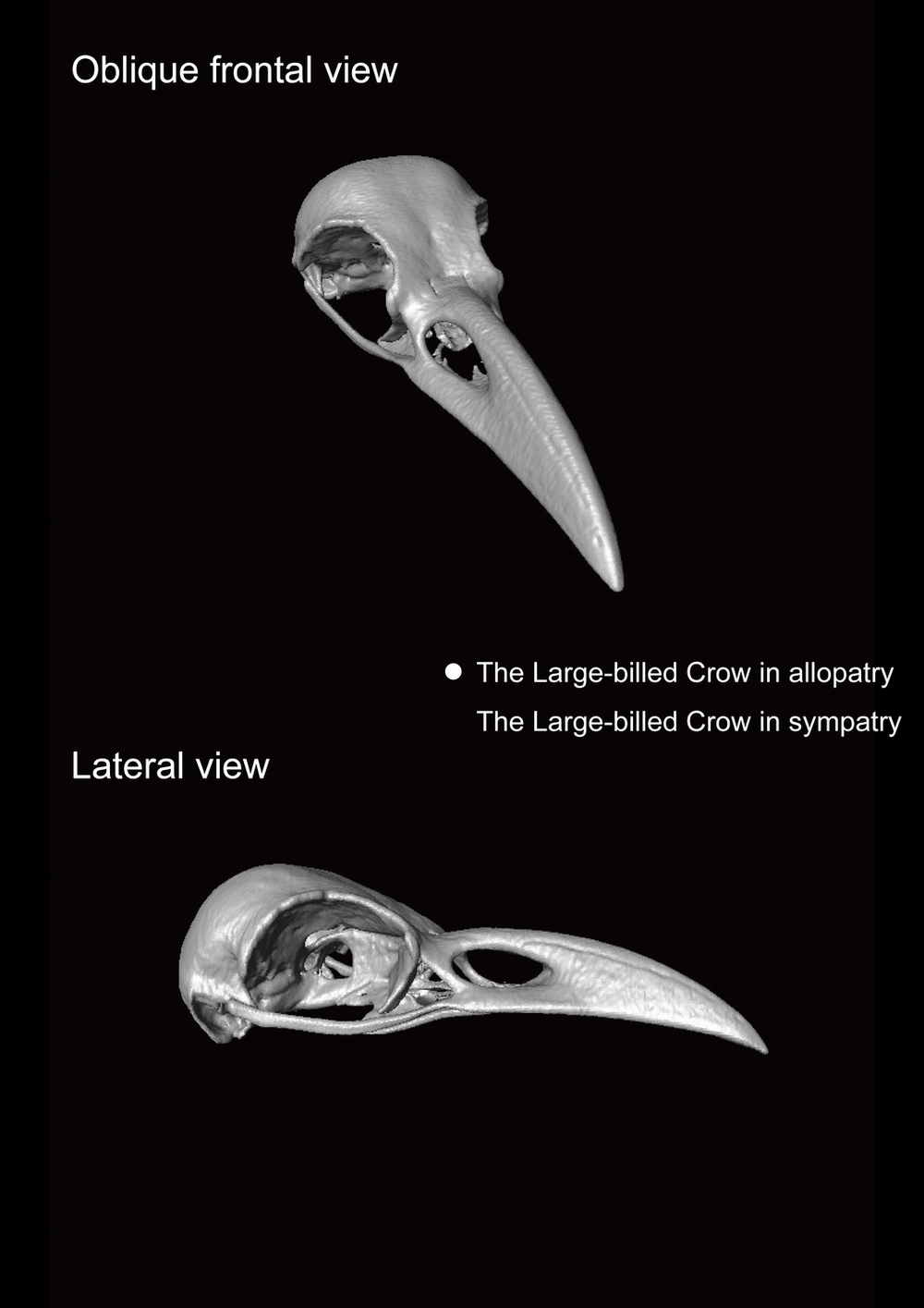

Supplement: Supplementary file 4 [file ECE3-8-10057-s004.gif]
